# Conductometric Sensors for Monitoring Degradation of Automotive Engine Oil^†^

**DOI:** 10.3390/s110908611

**Published:** 2011-09-05

**Authors:** Usman Latif, Franz L. Dickert

**Affiliations:** Department of Analytical Chemistry, University of Vienna, Waehringer Strasse 38, A-1090 Vienna, Austria; E-Mail: usman.latif@univie.ac.at

**Keywords:** conductometric sensors, engine oil, imprinted polymers, carbon nanotubes, polyurethane

## Abstract

Conductometric sensors have been fabricated by applying imprinted polymers as receptors for monitoring engine oil quality. Titania and silica layers are synthesized via the sol-gel technique and used as recognition materials for acidic components present in used lubricating oil. Thin-film gold electrodes forming an interdigitated structure are used as transducers to measure the conductance of polymer coatings. Optimization of layer composition is carried out by varying the precursors, e.g., dimethylaminopropyltrimethoxysilane (DMAPTMS), and aminopropyl-triethoxysilane (APTES). Characterization of these sensitive materials is performed by testing against oil oxidation products, e.g., carbonic acids. The results depict that imprinted aminopropyltriethoxysilane (APTES) polymer is a promising candidate for detecting the age of used lubricating oil. In the next strategy, polyurethane-nanotubes composite as sensitive material is synthesized, producing appreciable differentiation pattern between fresh and used oils at elevated temperature with enhanced sensitivity.

## 1. Introduction

Internal combustion engines in automobiles and other vehicles can only perform efficiently if there is proper lubrication between moving parts. Automotive engine oil is a mixture of base stock, and a number of additives which improve the operational properties of the lubricating oil [1]. Engine oils are susceptible to degradation by oxygen, increased temperature, and shear stress. The degradation of oil is very complex process and affects mainly additives and afterwards, oxidation of the base stock leads to formation of different acidic products, especially carbonic acids, and polymerization processes. The reduced lubrication capabilities caused by inadequate oil viscosity will damage the engine [2]. Thus, there is a need to design a sensor device which can tell us about chemical oil quality, by monitoring these directly in the oil. A variety of sensor devices have been proposed to monitor the degradation based on different parameters such as viscosity [3], conductivity [4], and acidity [5]. Further improvements were carried out by introducing molecular recognition properties in sensitive layers to determine the chemical quality of engine oil in gas or liquid phase by employing mass sensitive transducers [6–8]. Recently, dielectric spectroscopy has also been employed to detect the age of used engine oil [9]. In this paper, we have not only analyzed the electric properties of oil but molecular recognition properties were also introduced in functionalized polymers to differentiate between used oils on the basis of their conductance.

Molecular imprinting is a straightforward and universally adaptive technique to generate a recognition pattern in a polymer layer, which in combination with a suitable transducer leads to fast and label free sensor devices [10,11]. To produce recognition sites in the material, a model compound is added. This may be the analyte itself or any other suitable compounds owing to structure the material for reversible and selective inclusion of target analytes [12,13]. In polymerization, monomers engulf this template by a self-organization process, and after removal of the template, cavities are left behind which geometrically and chemically fit to the analyte. In the present work, we have templated the polymer layer with carbonic acid and coated it on an interdigital transducer, which proves to be very successful for measuring engine oil chemical quality. Insulating polymer materials can be converted into electrically conductive composites with the incorporation of conductive fillers, to form a three-dimensional conductive network [14,15]. Carbon nanotubes are composed of a hexagonal lattice of carbon atoms with one cylinder called as single walled carbon nanotubes (SWCNTs) or a multi-walled carbon nanotubes (MWCNTs) having concentric arrangements of many cylinders and can be grown up to 20 cm in length while having a radius of a few nanometers [16]. Multi-walled carbon nanotubes were discovered in 1991 [17], and single walled ones in 1993 [18,19]. Carbon nanotubes have extraordinary electrical and mechanical properties due to strong covalent bonding and show great promise for their use in many applications of nanotechnology [20–22]. Carbon nanotubes (CNTs), due to their outstanding electrical, mechanical, and anisotropic behavior, can serve to develop the next-generation polymer-based electrically conductive composites as conductive fillers [23]. Moreover, multi-walled carbon nanotubes are loaded on polyurethane as conductive filler to synthesize a sensitive layer for monitoring the age of lubricating oils.

## 2. Experimental Section

### 2.1. Chemicals

All the chemicals are obtained from Sigma Aldrich and ABCR in the highest available purity and used as received.

### 2.2. Synthesis of Titania Sol

Imprinted TiO_2_ sol-gel layer is prepared by dissolving 5 mg of template, *i.e.*, capric acid, in 400 μL of *iso*-propanol. Then, 67 μL of Ti(OBu)_4_ as a monomer is mixed into the solution. Afterwards, 10 μL of TiCl_4_ is added to initiate hydrolysis and condensation of the titanates. Water is not necessary for hydrolysis because the *iso*-propanol is not dried and contains 0.1% water, which is sufficient for hydrolysis. The above reaction mixture is heated at 60 °C for 1 h while stirring.

### 2.3. Synthesis of Silica Sols

Imprinted SiO_2_ sol-gel layers are synthesized by polymerizing functionalized alkoxy silanes. For optimization, polymer layers are generated by varying the monomers (dimethylaminopropyltrimethoxy silane (DMAPTMS), aminopropyltriethoxy silane (APTES), and diethylaminomethyltriethoxy silane (DEAMTES)), and then, dissolved it in 400 μL of ethanol along with 5 mg of capric acid as template. Then, 10 μL of water is added for hydrolysis. The mixture is stirred at 60 °C until the gelation point.

### 2.4. Synthesis of Polyurethane

The polyurethane layer is generated as follows: 197 mg of 4,4′-dihydroxy-2, 2-diphenylpropane (BPA) and 22.2 mg of 1,3,5-trihydroxybenzene (phloroglucinol) are dissolved in 500 μL of dry pyridine to make solution (A). Another solution (B) is prepared by dissolving 100 mg of 4,4′-diisocyanatodiphenylmethane (DPDI) in 500 μL of dry pyridine. Finally both the solutions A and B are mixed together and polymerization is carried out at 60 °C for 1 h in water bath.

### 2.5. Conductometric Measurements

**Figure 1.**
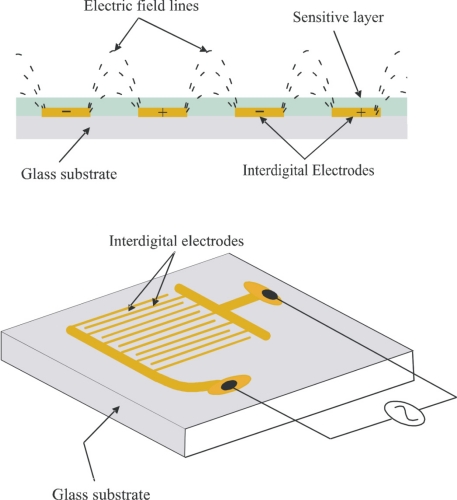
Schematic presentation of conductometric sensor and polymer layer covered interdigitated electrodes.

An interdigital electrode structure is fabricated on a glass substrate via screen printing with gold paste (GGP 2093126 W.C. Heraeus) and subsequently, covered with the sensitive layers. The thickness of the gold electrodes is measured by AFM, which is 171 nm. Interdigital transducers (IDTs) possess nine fingers in one electrode: the finger width is 300 μm, the gap 300 μm, and a length of 7 mm as shown in Figure 1.

**Figure 2.**
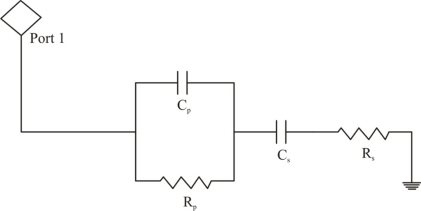
Equivalent circuit diagram of interdigital electrodes, covered with sensitive layer.

To investigate the properties of sensitive layer during conductometric measurements, the sensor is operated in an AC mode at an optimal working frequency by combining it with LCR meter (HP 4284 A) and data was sent to computer via a GPIB interface. The electric field lines are used to study the electric properties of the material. The equivalent circuit diagram of the interdigital electrode transducer is shown in Figure 2 [24], where resistance R_p_ and capacitance C_p_ are measured in parallel representing the electric properties of covered material on transducer. C_s_ and R_s_ are the serial capacity and resistance as represented by equivalent circuit diagram. Serial capacity is due to barriers between electrodes or layer, whereas, R_s_ is the resistance of contacts or wires.

To fabricate a sensor device, the sensitive layers of titania and silanes are spin coated on the interdigital transducer at a spinning rate of 2,500 rpm. Afterwards, these layers are dried in an oven and exposed to 5 μL volumes of different sample solutions at room temperature. AFM surface characterization enable us to determine the thicknesses of titania, dimethylaminopropyltrimethoxy silane (DMAPTMS), aminopropyl-triethoxy silane (APTES), and diethylaminomethyltriethoxy silane (DEAMTES) layers, which are 789, 1,098, 651, and 584 nm, respectively. The sensors are operated at a frequency of 20 Hz, while, their respective responses are read out by LCR meter. The sensor devices covered with titania, DMAPTMS, and DEAMTES sensitive layers are flushed with *n*-heptane, whereas, 0.1 M NH_4_OH in CH_3_OH is used for APTES to regenerate the sensor responses. In case of polymer-multiwalled carbon nanotubes (MWCNTs) composite, the nanotubes are dispersed in pre-polymer solutions and afterwards, 20 μL of the composite is drop coated on interdigital transducer. Multi-walled carbon nanotubes (MWCNTs) with diameter 6–20 nm and length of 1–5 μm are used to make a conductive linkage in polymer. Influence of different carbon nanotubes (CNTs) concentrations in composite are studied and best results are obtained with 10 wt%. This maximum amount of CNTs in polyurethane improves the electric behavior of polymer by forming a network. Polyurethane-CNTs composite device is used to study the quality of lubricating oil at 80 °C. For this purpose a customized setup is developed which contains an oil bath equipped with four containers. Three containers are filled with fresh oil, 40 h used oil, and 83 h used oil samples; whereas, a fourth one is filled with *n*-heptane to regenerate the sensor response. The measurements are carried out by dipping the sensor device in fresh and used oil samples and the respective responses are read out by LCR meter. In every case, the measurements are repeated for three times to test the reproducibility of sensor responses.

## 3. Results and Discussion

**Figure 3.**
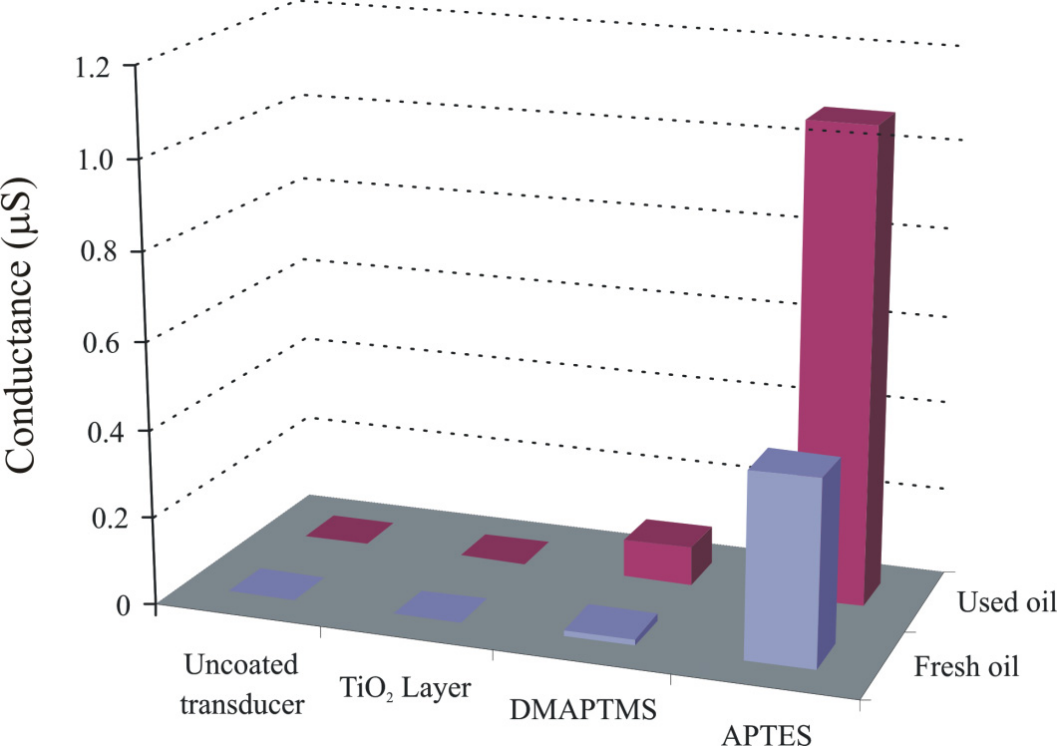
Normalized sensor responses of titania, dimethylaminopropyltrimethoxy silane (DMAPTMS), and aminopropyltriethoxy silane layers when exposed to fresh and 83 hours used oil samples. These responses are further compared with uncoated transducer behavior.

Engine oil is a very complex mixture of base stock and a number of additives. Inside automotive engines, the temperature is a basic factor for initiation of oxidation. Degradation of base oil involves the cracking of long chain hydrocarbons and subsequently, production of their respective acidic components. Capric acid is selected as a model compound for imprinting due to its stability and suitable length, allowing the detection of representative class of carboxylic acid compounds (C_5_–C_15_) [25]. Sol-gel layers [26] containing functional amino groups have proven very efficient for monitoring the acidic components produced during oxidation of engine oil [27,28]. As the sensitivity of the conductometric sensor depends on the polymer layer which is coated on the interdigital electrodes, in order to investigate the influence of layer components, two imprinted polymer layers from silicon alkoxide are synthesized by the sol-gel process containing different amino groups, whereas the third sensitive layer is produced from tetrabutoxyorthotitanate. The objective was to perform a stepwise optimization of layer composition to synthesize a polymer system with enhanced recognition properties. The pre-polymerized imprinted TiO_2_ and SiO_2_ solutions are spin coated on an interdigital transducer (IDT) at a spinning rate of 2,500 rpm respectively, to generate thin layers. The conductometric measurements are carried out at a frequency of 20 Hz and room temperature. Figure 3 shows the change in conductance of uncoated transducer, titania, dimethylaminopropyltrimethoxy silane (DMAPTMS), and aminoproplytriethoxy silane sensitive layers, by exposing to fresh and 83 h used oil samples. The thicknesses of these layers are 789, 1,098, and 651 nm, respectively.

The APTES layer shows pronounced sensitivity towards used oil in comparison to others, which is attributed to the presence of the amino groups as better interaction centers for the acidic components produced by degradation of engine oil, whereas in the case of DMAPTMS, the two methyl groups attached to amino groups suppress its interaction with analyte, which results in a reduced sensitive behavior.

**Figure 4.**
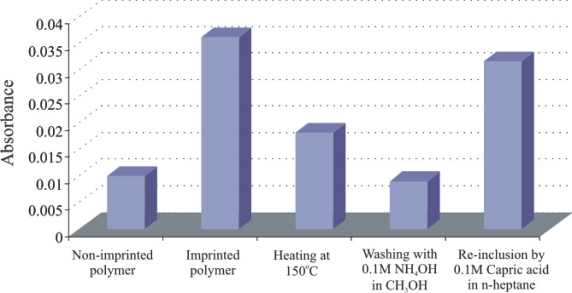
FTIR studies to monitor the removal and re-inclusion of capric acid in aminopropyltriethoxy silane polymer.

The recognition properties of silane layers mainly depend upon the amino groups. The surface characterization by AFM in contact mode reveals that there is no significant difference in the porosity of DMAPTMS and APTES layers. The APTES layer strongly interacts with the analyte and acts like a dosimeter, while, it is preferably removed by washing with 0.1 M NH_4_OH in CH_3_OH (in comparison to other sensitive layer which are regenerated by *n*-heptane) and FT-IR measurements are performed to monitor the removal and re-inclusion of capric acid in sol-gel layer. The changes in stretching and anti-stretching vibrations of the methylene groups at 2,925 cm^−1^ are observed via IR spectroscopy. The samples are analyzed on a *Perkin Elmer System 2000 FT-IR Spectrophotometer* using an ATR (attenuated total reflection) cell. A volume of 5 μL of each sample (imprinted and non-imprinted) is spin coated on a glass slide at a spinning rate of 2,500 rpm and exposed to a diamond crystal of an ATR cell soon after drying (at room temperature), heating (150 °C), washing (0.1 M NH_4_OH in CH_3_OH), and re-inclusion (0.1 M capric acid in heptanes). The respective behavior of layer is shown in Figure 4, which strongly supports the presence and subsequently, removing of capric acid by heating and washing. The removal leaves behind adapted cavities for re-inclusion of capric acid. To verify this, layer is kept in 0.1 M capric acid solution in *n*-heptane. The results thus show an increase in signal indicating the re-inclusion of capric acid in the ceramic sol-gel layer.

**Figure 5.**
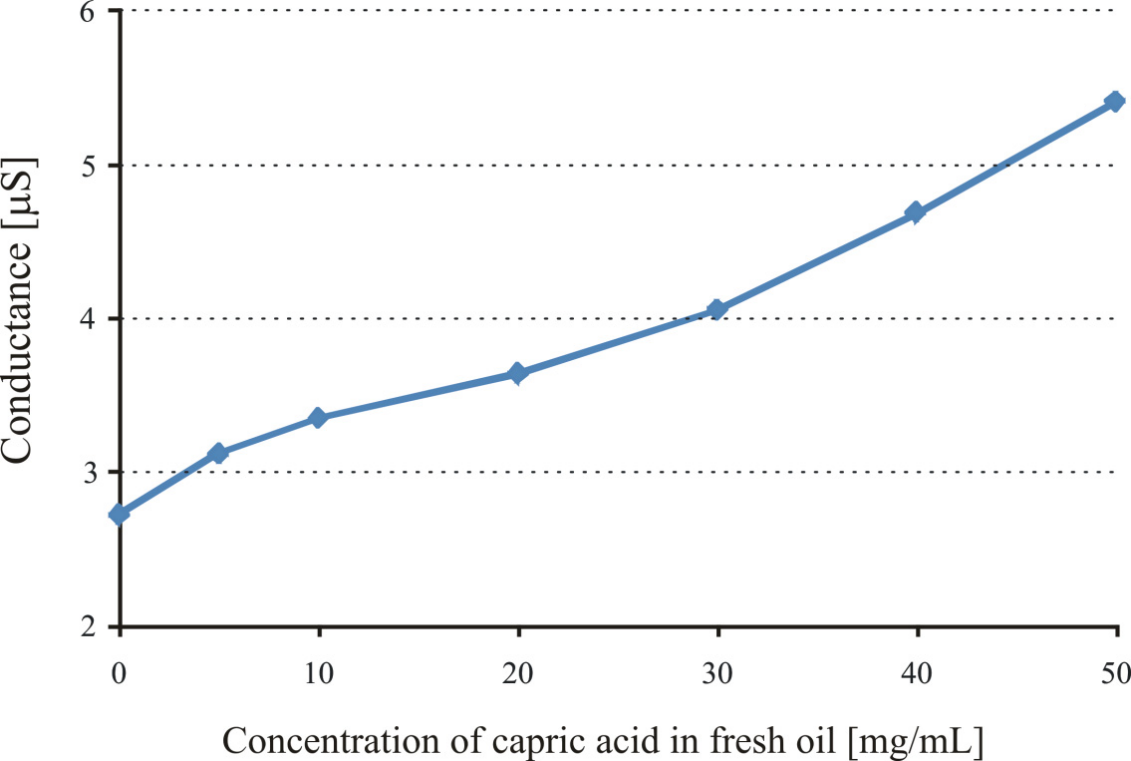
Sensor responses of imprinted polymer layer (APTES) while exposing to fresh oil with different concentrations of capric acid.

The acidic products are directly linked to the oxidation of lubricant or more precisely the age of the engine oil, so we use capric acid as a model compound and prepare different concentrations of it in fresh oil. The imprinted layer (APTES) is exposed to oil with different concentrations of capric acid and the observed sensor responses are shown in Figure 5.

**Figure 6.**
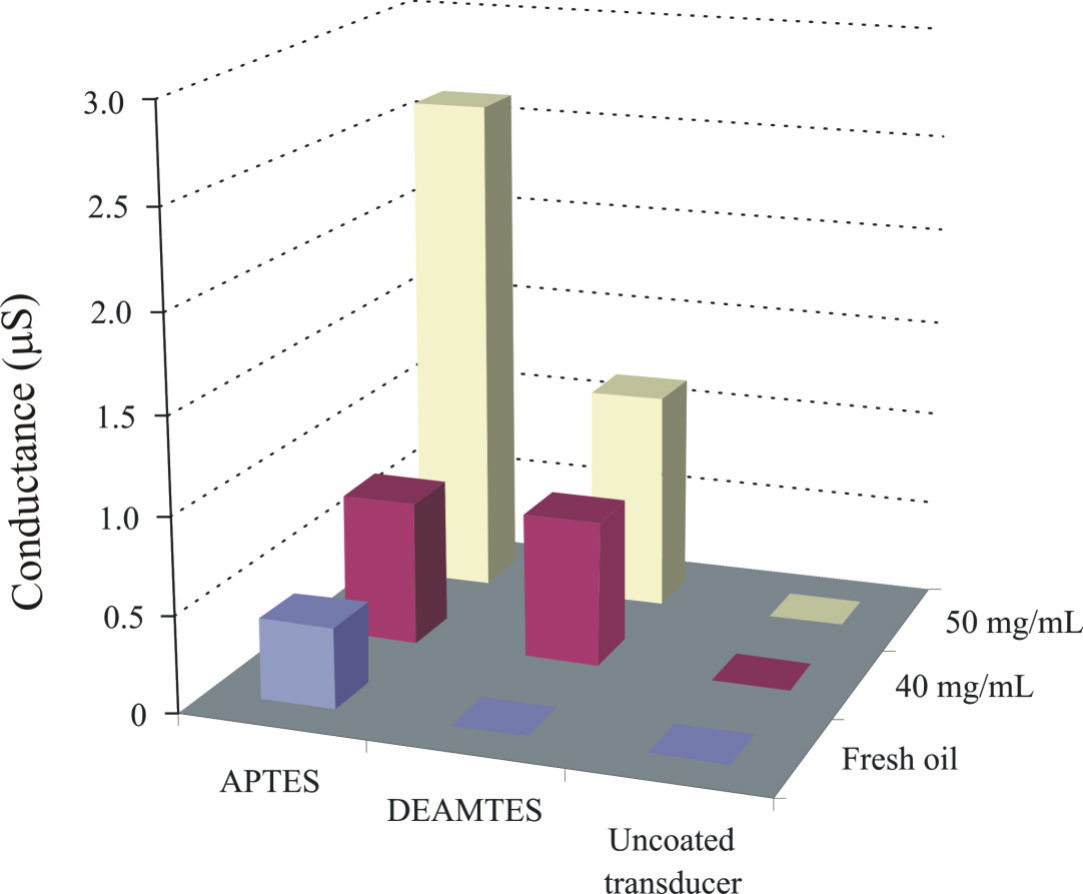
Normalized sensor responses of DEAMTES and APTES layers, while, exposing to 40 mg or 50 mg ammonium salt of capric acid in fresh oil, at room temperature and 20 Hz frequency. These responses are compared with uncoated interdigital transducer behavior.

The capric acid imprinted APTES layer shows a conductance of about 2.7 μS. Recognition layer is highly sensitive over a wide concentration range of capric acid in fresh oil, which shows the successful imprinting effects. The sensitivity behavior of the polymer also depends on the amino groups, which interact with the acidic components present in the exposed samples. Pronounced sensor responses are observed with increasing concentration of capric acid in fresh oil. Inside automotive engines, the temperature is quite high and can initiate the oxidation of base oil components. This involves the cracking of long chain hydrocarbons or their polymerization and production of their respective acidic materials. Some basic materials are added to oil to neutralize these acidic components for better performance of the engine oil and to prevent corrosion of metal parts. Thus, the amount of salts present in used engine oil directly links to the acidic products and provides us information about the age of used oil. The sensitivity of the APTES layer towards different percentages of capric acid has already been highlighted, now another aminosilane polymer (DEAMTES) is introduced which has only one methylene group between nitrogen and silicon in order to see the effect of smaller chain length on interaction behavior. The thicknesses of these polymers are 651, and 584 nm, respectively. The behavior of the DEAMTES layer is similar to that of APTES, but it shows less conductance. The salt solutions of ammonium caprate in fresh oil are used to evaluate the interaction mechanism of sensitive layers, as shown in Figure 6.

**Figure 7.**
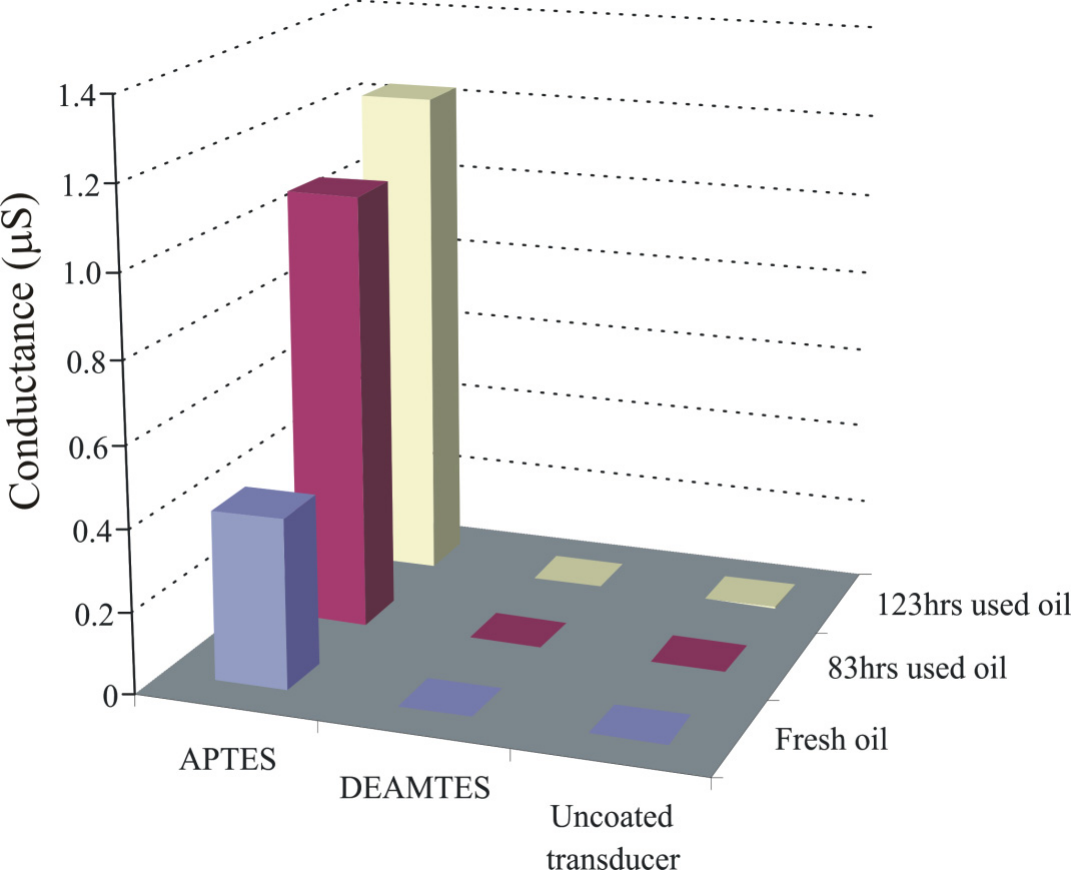
Assessing the quality of engine oil by conductometric sensors covered with DEAMTES, and APTES layers and compared with uncoated interdigital transducer behavior.

Appreciable effects are observed in case of the APTES layer, whereas uncoated transducer shows no effect of electrolytes in the bulk solutions. There may be two possibilities to explain the irresponsive behavior of an uncoated transducer: (1) hydration of ammonium salts will produce carbonic acid, ammonia, and water which will not appreciably contribute to changing the conductance of the uncoated interdigital transducer. (2) A contact ion pair, which is formed in a medium with low dielectric constant, leads to no conductance change. In any case, the change in conductance is contributed to the proposed coated materials when exposed to different concentrations of salt solutions. Thus, we can say that observed sensor responses are due to changes in the conductance of sensitive layers when exposed to the analyte solutions. The response of APTES is much higher than that of the DEAMTES sensitive layer because in APTES the amino group is totally exposed to the analyte while in DEAMTES two ethyl groups suppress its interaction and diminishing the carbon chain could not enhance the recognition behaviour of DEAMTES layer. Thus, an APTES sensitive layer provides maximum interaction sites to the analyte in a sterically unhindered way which is detected by a conductometric sensor. The behavior of these aminosilanes is further analyzed against different oil samples, as shown in Figure 7.

This picture shows the normalized sensor response of coated materials. The solvent *n*-heptane is used for regeneration of the DEAMTES coated sensor device, whereas, for APTES 0.1 M NH_4_OH in CH_3_OH is applied. The APTES layer shows a strong interaction towards the degradation products in used lubricating oil in comparison to DEAMTES, due to the presence of amino groups which are totally exposed, whereas, in DEAMTES two bulky ethyl groups suppress its interaction behavior. The graph shows that the APTES conductometric sensor can successfully monitor the degradation of engine oil in comparison to the DEAMTES layer. A composite material from titania and aminopropyltriethoxy silane was also synthesized and exposed to fresh and used oil samples. Similar differentiation pattern was observed, but with less sensor response.

**Figure 8.**
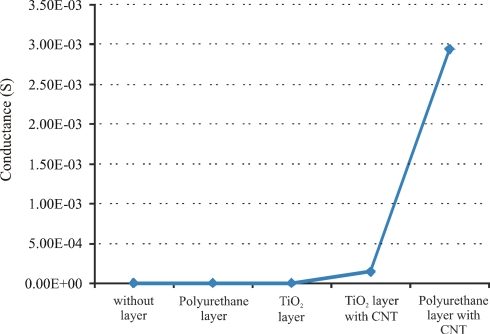
Conductance of titania and polyurethane layers with and without multi-walled carbon nanotubes.

In the next strategy, multi-walled carbon nanotubes-polymer (titania and polyurethane) composites are synthesized and coated on the interdigital transducer to fabricate a sensor system. Multi-walled carbon nanotubes (MWCNTs) with diameter 6–20 nm and length of 1–5 μm are used to make a conductive linkage in the polymer. A small amount of pre-polymerized TiO_2_ and polyurethane is coated on separate interdigital electrodes and dried at 110 °C for 2 h in an oven. After drying, the resulting layers show excellent adhesion to the substrate and did not peel off. For the fabrication of nanotubes-polymer composites on interdigital electrodes MWCNTs (10 wt%) are dispersed in small amounts of polymer solutions to make a suspension and then, drop-coated on IDE to make a highly concentrated conducting links by bridging the finger gaps. After coating, the nanotubes-polymer composites are dried in an oven at 110 °C for 2 h. The conductance of all these fabricated sensors is measured at a frequency of 20 Hz, as shown in Figure 8.

**Figure 9.**
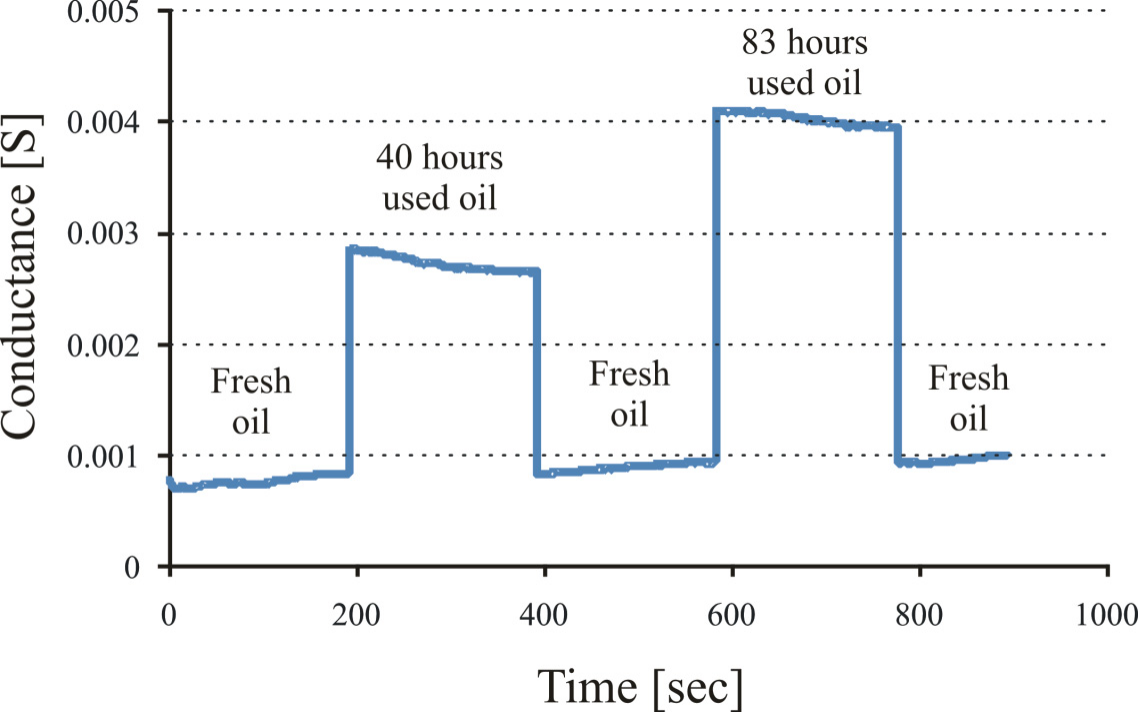
Conductance of polyurethane-nanotubes composite while exposing to fresh and used oil samples at 80 °C.

The conductance of these sensitive layers is measured at room temperature. The insulating polymers (without carbon nanotubes) show very low conductance, while, polymers with conductive filler (carbon nanotubes) present higher conductance values. Nanotubes-polyurethane composite shows a better conductivity than titania composite because of the thickness of the resulting polymer. The sensitive layer of titania polymer is very thin and does not accommodate many CNTs, while, in the case of polyurethane polymer, it forms a thicker layer than titania, and can accommodate maximum CNTs to make concentrated conductive links. The improvement of electrical behavior of nanotubes composite polymer depends upon the amount of conductive fillers. The influence of different CNT concentrations in the composite are investigated and the best results are obtained with 10 wt%. This maximum amount (10 wt%) of carbon nanotubes in polyurethane represents the formation of an enhanced nanotubes network. The behavior of the above mentioned conductometric sensor (polyurethane-nanotubes composite) is investigated against fresh and used oils at 80 °C, as shown in Figure 9. These measurements are carried out by using a homemade apparatus. It consists of an oil bath equipped with four containers. Three containers contain the samples of fresh oil, 40 h used oil, and 83 h used oil, whereas, the fourth one is filled with *n*-heptane. These samples are heated to 80 °C, then exposed to conductometric sensor respectively and *n*-heptane is used for washing between measurements. The kinetics of this result reveals that change in conductance of sensor system is a surface phenomenon, which is attributed to charge transfer between oxidized products in the used lubricating oil and carbon nanotubes. The data acquisition was stopped during sensor regeneration and time of change of analyte but the rise time and recovery is short in comparison to the plateau.

Due to these functionalities of the sensor, it can be used for online monitoring of engine oil degradation. The conductometric sensor successfully differentiates between fresh and used oil samples at elevated temperature (80 °C), while dipping it in solution. So, by the use of this sensor, the age of the oil in automotive engines can be determined, which will indicate the exact time for oil-changes. Carbon nanotubes show excellent electrical properties due to their sp^2^ bonding. Moreover, their electrical conductance can be further increased by the incorporation of hydrogen peroxide [29]. The degradation of engine oil is attributed to free radical chain reaction caused by mechanical shear stress or due to transfer of energy in the form of heat which relates to temperature of combustion engines. As a result, free radicals are produced from the cleavage of hydrocarbon molecules, which will react with oxygen to form alkyl peroxy radicals and further hydroperoxides, alkyl radicals in a second step [30]. These oxidation processes continue and result into the production of carbonic acids. The main sensing mechanism is the charge transfer between oxidized components and carbon nanotubes channels. The result shows the electron withdrawing effect of carboxylic acids making the conductivity of the composite relatively higher. This effect was also observed while monitoring the oxidation of edible oils by using carbon nanotubes [31]. The oxidation products of edible oils are mainly polar compounds, such as alcohols, aldehydes, ketones, and carboxylic acids. These oxidation products withdraw electrons from nanotubes and cause an increase in the conductance of sensor. This phenomenon was further confirmed by adding oleic acid to fresh oil. At first, the sensor was exposed to fresh edible oil which results in an increase in the resistance of the sensor and after the addition of oleic acid, the response of the sensor decreased slightly. The finding shows the electron withdrawing effect of oleic acid. We also have observed similar effects when adding capric acid to fresh oil. The conductance of the sensitive layer increases with increasing concentration of capric acid in fresh oil. In the present case, used lubricating oil samples are exposed to the sensor alternatively with fresh oil. Of course, the acidic components increase with oil age due to degradation of base oil as characterized by infrared (IR) spectroscopy [32]. Consequently, this sensor can determine the condition of engine oils by difference in responses of carbon nanotubes. Conductance of nanotubes-polymer composite depends upon the amount of conductive fillers and the thickness of the layer. In the previous experiment, a 20 μL suspension of CNTs (10 wt%) in polyurethane was drop-coated. The conductance of such layers is enhanced by increasing the thickness by coating 100 μL polyurethane-CNTs composite. The electrical properties are modified by the evaporation of solvent. A change in the conductivities of the composites by seven orders of magnitude is observed in parallel to the variation of nano-tube lengths from 5 μm to 200 μm. Further detection principles are obtained by oxidized nanotubes. Optimistic perspectives are revealed by comparing the carboxylic acid derivatives and their neutralized products.

## 4. Conclusions

Interdigital electrode structures are used as transducers and combined with imprinted polymers to design a conductometric sensor for monitoring the age of used lubricating oil. Titania and silica imprinted polymers are synthesized by following a sol-gel strategy and used as sensitive coatings. Optimization of silica layers is performed by varying the monomers such as dimethylaminopropyltrimethoxy silane (DMAPTMS), aminopropyltriethoxy silane (APTES). Characterization of recognition materials are carried out by measuring the change in conductance of these layers while exposed to different concentrations of carbonic acid and its ammonium salt in fresh oil. The measurements reveal that silica layer in which amino group is totally exposed is a promising candidate for detecting the quality of engine oil, which is attributed to its unhindered interaction with acidic components present in used lubricating oil, whereas, in the other sensitive layer two bulky groups (ethyl or methyl attached to the amino group) suppress the recognition properties. Moreover, polyurethane layers loaded with multi-walled carbon nanotubes as conductive fillers enable us to monitor the quality of engine oil with enhanced sensor responses. The electrical conductance of carbon nanotubes increases with the oxidation products produced due to the degradation or more precisely with the age of oil in internal combustion engines. Layer conductance remains almost the same when exposed to fresh oil samples and changes only with the incorporation of oxidized products in the lubricating oil. Thus, in every measurement, conductance of fresh oil is considered as the initial state of the sensor system. A correlation between age of oil and their respective intensities has already been published by our group, while pursuing the spectroscopic analysis of degraded engine oils [32]. In every case the measurements are repeated three times and reproducibility is observed. In this case, we focused on the sensor phenomenon and we will test the long term stability of the sensor in the future.
